# Diseases of Children

**Published:** 1890-09

**Authors:** DeLancey Rochester


### DISEASES OF CHILDREN.

By DeLANCEY ROCHESTER, M. D.

DESSAN, S. HENRY, M. D., (*Archives of Pediatrics*, vol. vii., pp. 600, *et seq.*,) Antipyrin in Diseases of Children, calls attention to the fact "that no class of remedies of recent introduction has proved of so much value and interest to the medical practitioner as that of which antipyrin is the type," and that "especially is this statement applicable to the diseases of infancy and childhood."

It is more as a nervous sedative than as an antipyretic, however, that it is useful among children.

In the pneumonias of children he gives it for its double effect when the temperature reaches 104° F. or over. He administers it in two and a half to five grain doses, repeated every hour for four doses, once in the twenty-four hours, preferably towards evening, "so as to secure sleep, which commonly follows as a result of its sedative action."

His "most marked success with antipyrin has been in the treatment of chorea." He has treated, in all, seven cases, "two being still under treatment, with improvement. Of these seven cases, one was cured in one week, two in three weeks, one" (in which arsenic bromide of potassium, iron and digitalis had failed) "in four weeks, and one, the severest of all, in six weeks." "When we reflect that according to the report of the collective investigating committee of the British Medical Association on chorea . . . . the results of drug treatment, the favorite remedies being arsenic and iron, showed an average duration for the disease of ten weeks, the same duration being shown with non-drug treatment (that is hygienic and dietetic measures only), I think we are warranted in granting a foremost place to antipyrin in the management of chorea. The average duration for the disease in my own cases was only four weeks."

He also mentions favorably its already well-known action as a remedy in acute articular rheumatism, and in pertussis. He has found a new use for antipyrin in the treatment of "cases of *urticaria* that have resisted the time-honored treatment of rhubarb and soda mixture, either alone or combined with bromide of potassium or many other remedies." He says: "Let it be distinctly understood that I do not refer to ordinary acute attacks of urticaria, that will disappear under restricted diet alone, but the persistent cases that will continue in an intermittent manner for months in spite of arsenic and all other known remedies. In such cases I have found antipyrin to act with speedy relief."

He also states that he has had several cases of headache and neuralgia in children "that have been relieved promptly as in adults."

### THE USE OF COMMERCIAL MILK SUGAR IN INFANT FEEDING.

Brush, E. F., M. D., (*Archives of Pediatrics*, vol. vii., pp. 605, *et seq*.,) read an article with this title, before the American Medical Association at Nashville, in which he shows that commercial sugar of milk, that is now so much used in infant feeding, is an unreliable article as to purity; and, even when pure, has different *physiological* properties from those of the sugar as it exists in human milk. He says:

One of the faults of physiological chemists is that they make no distinction between a substance existing in a natural condition and the substance eliminated and isolated by chemical means. Thus, the sugar of milk of commerce and the sugar of milk as it exists in that fluid are regarded by the chemist as one and the same thing. Hence, the physician has been led into the error of thinking that as the sugar of milk is that designed by nature as the best saccharine nutrient, therefore the isolated sugar must fulfil the same function. This is not the truth. Sugar of milk in that fluid is all assimilated, and the milk-sugar of commerce, when added to baby food, is eliminated both by the kidneys and by the bowels. This I have demonstrated by numerous experiments. I have never found sugar present in the urine or feces of babies fed at the breast, but in three cases of infants fed with mixtures containing milk-sugar to the amount of three ounces or more, in twenty-four hours I have always found sugar in the urine and feces, demonstrated by Fehling's test. . . . . . Therefore, instead of being of value as a nutrient, it must be harmful, to what extent I am not at present prepared to say. A substance that is not broken up in the system, but eliminated without change, if it be not an absolute poison, will produce little, if any, appreciable immediate effect.

These remarks of Dr. Brush's are interesting, as they show that the problem of infant feeding has not, even theoretically, as it certainly has not practically, been solved by Roche's modification of Meigs's mixture; but it is a pity that Dr. Brush has not completed his observations. It is possible that there may be a difference between the sugar of cow's milk and the sugar of human milk, as there certainly is a difference in the case of the two milks that is something more than a greater or less dilution by water.

In the present status of our knowledge, we must be thankful that there are so many different food preparations possible for the nourishment of the infant, for of no class of beings is it more true that "what is one's meat is another's poison."

